# Low Levels of Low-Density Lipoprotein Cholesterol and Mortality Outcomes in Non-Statin Users

**DOI:** 10.3390/jcm8101571

**Published:** 2019-10-01

**Authors:** Ki-Chul Sung, Ji Hye Huh, Seungho Ryu, Jong-Young Lee, Eleonora Scorletti, Christopher D Byrne, Jang Young Kim, Dae Sung Hyun, Sang-Baek Ko

**Affiliations:** 1 Division of Cardiology, Department of Internal Medicine, Kangbuk Samsung Hospital, Sungkyunkwan University School of Medicine, Seoul 03181, Korea; jyleeheart@naver.com; 2 Division of Endocrinology and Metabolism, Department of Internal Medicine, Wonju Severance Christian Hospital, Yonsei University Wonju College of Medicine, Wonju 26426, Korea; 3 Department of Occupational and Environmental Medicine, Kangbuk Samsung Hospital, Sungkyunkwan University School of Medicine, Seoul 03181, Korea; sh703.yoo@gmail.com; 4 Nutrition and Metabolism Unit, IDS Building, Southampton General Hospital, (University of Southampton) and Southampton National Institute for Health Research Biomedical Research Centre, Southampton SO17 1BJ, UK; e.scorletti@soton.ac.uk (E.S.); c.d.byrne@soton.ac.uk (C.D.B.); 5 Department of Gastroenterology, University of Pennsylvania, Perelman School of Medicine, Philadelphia, PA 19104, USA; 6 Division of Cardiology, Department of Internal Medicine, Wonju Severance Christian Hospital, Yonsei University Wonju College of Medicine, Wonju 26426, Korea; kimjy@yonsei.ac.kr; 7 Department of Preventive Medicine and Institute of Occupational and Environmental Medicine, Yonsei University Wonju College of Medicine, Wonju 26426, Korea; hyonds@hanmail.net (D.S.H.); kohhj@yonsei.ac.kr (S.-B.K.)

**Keywords:** low density lipoprotein cholesterol, mortality, cancer, cardiovascular disease

## Abstract

We aimed to test the association between low-density lipoprotein cholesterol (LDL-C) and cardiovascular disease (CVD), cancer, and all-cause mortality in non-statin users. A total of 347,971 subjects in Kangbuk Samsung Health Study (KSHS.57.4% men, mean follow up: 5.64 ± 3.27 years) were tested. To validate these associations, we analyzed data from another cohort (Korean genome and epidemiology study, KoGES, 182,943 subjects). All subjects treated with any lipid-lowering therapy and who died during the first 3 years of follow up were excluded. Five groups were defined according to baseline LDL-C concentration (<70, 70–99, 100–129, 130–159, ≥160 mg/dL). A total of 2028 deaths occurred during follow-up in KSHS. The lowest LDL-C group (LDL < 70 mg/dL) had a higher risk of all-cause mortality (HR 1.95, 1.55–2.47), CVD mortality (HR 2.02, 1.11–3.64), and cancer mortality (HR 2.06, 1.46–2.90) compared to the reference group (LDL 120–139 mg/dL). In the validation cohort, 2338 deaths occurred during follow-up. The lowest LDL-C group (LDL < 70 mg/dL) had a higher risk of all-cause mortality (HR 1.81, 1.44–2.28) compared to the reference group. Low levels of LDL-C concentration are strongly and independently associated with increased risk of cancer, CVD, and all-cause mortality. These findings suggest that more attention is needed for subjects with no statin-induced decrease in LDL-C concentrations.

## 1. Introduction

For decades, low-density lipoprotein cholesterol (LDL-C) has been considered to be the major causative factor in the development of atherosclerotic cardiovascular disease (CVD) and CVD mortality [1]. Numerous studies have robustly represented that reduction of plasma LDL-C concentration by lipid lowering agents is associated with a greater reduction in development of CVD and CVD mortality [2,3,4,5,6,7,8,9,10].

In contrast to the enormous evidences from previous studies regarding CVD, the correlation between low plasma concentrations of LDL-C and mortality outcome is still uncertain especially in relatively healthy populations. In most randomized controlled trials or observational studies, subjects with unusually low concentration of LDL-C level have been excluded in analysis. Therefore, to date, we could not clearly find the impact of lower LDL-C on mortality outcome, especially in subjects who does not take lipid lowering agents. Moreover, some recent Japanese epidemiological studies have shown that high total cholesterol is not a risk factor for CVD and it is rather conversely associated with overall mortality [11]. Similarly, other observational study showed that healthy individuals with low LDL-C have a significantly increased risk of both infectious diseases and cancer [12,13]. These studies raised an important issue whether low level of LDL-C could be related to all-cause mortality and cancer mortality in healthy populations. However, no study has evaluated the impact of LDL-C, not statin-induced decrease in LDL-C concentrations, on all-cause, cancer, and CVD mortality.

Since the effect of low concentrations of LDL-C on cancer and overall mortality remains controversial, we have investigated the associations between low levels of serum LDL-C, and cancer, all-cause mortality, and even CVD mortality in a very large, young, and well characterized, relatively healthy occupational cohort (Kangbuk Samsung health study, KSHS) during a median 5.82-year follow-up. To validate these associations, we then analyzed other dataset from a large population-based cohort study with government funding, named the Korean genome and epidemiology study (KoGES).

## 2. Methods

### 2.1. Study Population

The study population consisted of individuals who participated in a comprehensive health screening program with serum LDL-C at Kangbuk Samsung Hospital, Seoul, Korea from 2002 to 2012 (*n* = 396,951). The purpose of the screening program was to promote health through early detection of chronic diseases and their risk factors. Additionally, the Korean Industrial Safety and Health Law demands working individuals participate in an annual or biennial health examination. For this analysis, subjects were excluded for one or more of the following reasons: Subjects with missing data for smoking, alcohol, exercise, or lipid profiles at baseline (*n* = 42,020); subjects with lipid medication (*n* = 3667); subjects with histories of malignancy (*n* = 5342); subjects with mortality within 3 years after baseline (*n* = 649). Some of the excluded subjects had more than one of the above exclusion criteria. The total number of eligible subjects for testing associations with all-cause and CVD mortality was 347,971 (median follow up: 5.82 (IQR 2.62–8.63) years and mean (SD) follow up: 5.64 (±3.27) years). This study was approved by the Institutional Review Board of Kangbuk Samsung Hospital. Requirement for informed consent was waived as de-identified information was retrieved retrospectively.

In the validation cohort, the cohort profile of KoGES has been previously reported [14]. The KoGES cohort was designed to investigate and assess genetic and environmental factors as correlates or determinants of the incidence of chronic diseases, (e.g., type 2 diabetes, hypertension, CVD, and cancer) in Koreans. The number of baseline subjects was 211,714. For this analysis, subjects were excluded for one or more of the following reasons: Subjects with missing data for smoking, alcohol, exercise or lipid profiles at baseline (*n* = 4149); subjects with lipid medication (*n* = 16,488); subjects with histories of malignancy (*n* = 6578); and subjects with mortality within 3 years after baseline (*n* = 1556). The total number of eligible subjects for testing associations with all-cause and CVD mortality was 183,943 (mean (SD follow up: 8.57 (±2.59) years). The percentile of women was 65.4%. At each visit, informed written consent was obtained from all participants. The study protocol was approved by the Ethics Committee of the Korean Center for Disease Control and the Institutional Review Boards of Yonsei University Wonju College of Medicine.

### 2.2. Data Collection

As part of the health screening program, individuals completed questionnaires related to their medical and social history and medication use. Individuals were asked about duration of education (years), frequency of exercise (none, less than once a week, at least once a week, ≥3 times per week (regular exercise)), smoking history (never, former, or current) and alcohol consumption (grams (g)/week). Trained staff also collected anthropometric measurements and vital statistics. Body weight was measured in light clothing with no shoes to the nearest 0.1 kg using a digital scale. Height was measured to the nearest 0.1 cm. Body mass index (BMI) was calculated as weight in kilograms divided by height in meters squared. Blood pressure was measured using standard mercury sphygmomanometers. Blood samples were collected after minimum 10 h of fasting and analyzed in the same core clinical laboratory. The core clinical laboratory has been accredited and participates annually in inspections and surveys by the Korean Association of Quality Assurance for Clinical Laboratories. Serum levels of total cholesterol, triglycerides, LDL-C, and high-density lipoprotein cholesterol (HDL-C) were measured using Bayer Reagent Packs (Bayer Diagnostics, Leverkusen, Germany) on an automated chemistry analyzer (Advia 1650 Autoanalyzer; Bayer Diagnostics, Leverkusen, Germany).

Deaths among participants were identified by matching the information to death records from the National Statistical Office using identification numbers assigned to subjects at birth. Causes of death were coded centrally by trained coders using the ICD-10 classification (International Classification of Diseases, 10th revision). In this study, CVD mortality was defined as ICD-10 codes I00 to I99.

### 2.3. Statistical Analysis

The statistical analysis was performed using STATA version 14.0 (StataCorp LP, College Station, TX, USA). Reported *p* values were two-tailed, and <0.05 were considered statistically significant. The distribution of continuous variables was evaluated and transformations were conducted for nonparametric variables. We divided our subjects according to plasma LDL-C concentrations (<70, 70–99, 100–129, 130–159, ≥160 mg/dL) at baseline. Cox proportional hazards models stratified by five groups were used to estimate hazard ratios (HRs and 95% CIs for all-cause mortality, CV, and cancer mortality in each LDL-C category, compared with the LDL-C 100–129 mg/dL as the reference group). This LDL-C 100-129 mg/dL group was chosen as the reference because this group contained the mean LDL-C concentration for adults in Korea over the last 10 years (approximately 110 mg/dL) [15]. For testing linear risk trends across LDL-C concentration groups in the regression models, we used the categories rank as a continuous variable. To minimize the influence of possible “reverse causation” (illnesses causing low LDL-C), we excluded the subjects who died with in less than 3 years after the baseline measurements. A cubic spline analysis was used to characterize non-linear, dose-response associations between LDL cholesterol levels and mortality, and to minimize residual confounding for continuous confounders [16,17]. We checked the proportional hazards assumption by examining graphs of estimated log (-log) survival. *p* < 0.05 was considered significant.

## 3. Results

### 3.1. Baseline Characteristics of Participants

A total of 347,971 subjects (mean age 39.6 years) (57.4% men) were studied in KSHS over a mean follow up of 5.64 ± 3.27 years. Five groups were defined according to the level of baseline LDL-C concentration (<70, 70–99, 100–129, 130–159, ≥160 mg/dL). Table 1 shows baseline characteristics of study participants respectively according to LDL-C concentrations at baseline. Subjects in the lowest LDL-C group (LDL < 70 mg/dL) were of similar age to subjects in each of the other groups with a small albeit significant increase in age from the lowest to the highest LDL-C groups. Blood pressure was also very similar across LDL-C groups with a small albeit significant increase in systolic BP from the lowest to the highest LDL-C groups. Systolic BP was −2 mmHg lower in the lowest LDL-C group compared with the highest LDL-C group. The proportion of current smokers and former smokers was remarkably similar across LDL-C groups.

### 3.2. Association between Low-Density Lipoprotein Cholesterol and All-Cause, CVD, and Cancer Mortality in Kangbuk Samsung Health Study

**Figure 1:**
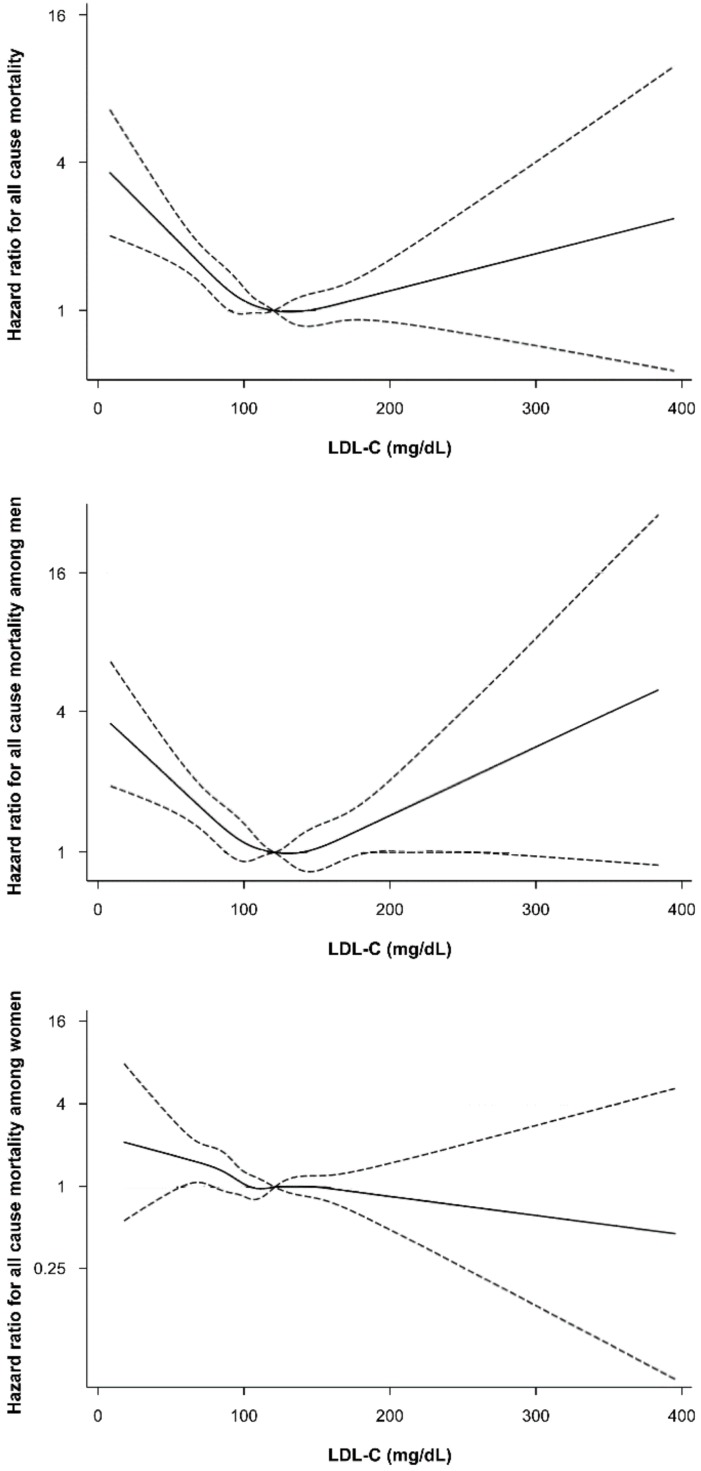
Spline plot of plasma low-density lipoprotein cholesterol and all-cause mortality rate, normalized to the mortality rate at low-density lipoprotein cholesterol of 100–129 mg/dL. The adjusted hazard ratios and 95% confidence intervals were calculated with Cox proportional-hazards models after adjusting for adjusted for age, BMI, smoking status, alcohol intake, regular exercise, education, house income, marital status, diabetes, and hypertension, and high-density lipoprotein cholesterol.

There were 1379 deaths (0.40%) during follow up in KSHS cohort (Mean age of death: 58.2 (13.8). Of these deaths, 675 deaths were due to cancer, (482 deaths in men and 193 deaths in women). There were 188 deaths from CVD (134 in men and 54 deaths in women). The results of cox regression models showing risk of all-cause mortality, CVD mortality, and cancer mortality according to baseline LDL-C concentrations are shown in Table 2, Table 3 and Table 4, respectively. Inspection of spline plots revealed U-shaped association between LDL-C concentrations and all-cause mortality, in which mortality risk increases significantly with LDL-C less than 70 mg/dL in men (Figure 1). A higher risk of all-cause mortality was observed in lowest LDL-C group compared with the LDL-C 100–129 mg/dL as the reference group (Table 2). After adjusting for age, BMI, smoking status alcohol intake, regular exercise, educational level, history of hypertension, diabetes, and CVD, and HDL-C concentration, the association between the lowest LDL-C levels and higher risk of all-cause mortality still remained significant (HR 1.95 [1.55–2.47]). However, this association was significant only in men (HR 2.07 [1.58–2.70] for men; HR 1.56 [0.95–2.55] for women). The lowest LDL-C group (LDL < 70 mg/dL) was also associated with increased risk of CVD mortality compared to the reference group (Table 3). When we stratify subjects by sex, we observed the highest risk of CVD mortality was seen in both men and women, although the statistically significance slightly attenuated due to low incidence of cardiovascular mortality (HR 1.99 (0.99–4.02) for men, HR 2.41 (0.80–7.29) for women). Similarly, the lowest LDL-C levels were significantly associated with higher risk of cancer mortality (HR 1.81 (1.44–2.28)) and this association was more prominent in men than in women (Table 4). Since there was a slightly higher proportion of subjects with diabetes or with a history of CVD in the lowest LDL-C, all the regression models were repeated after exclusion of these subjects in order to ensure the robustness of the results (Tables S1–S3). The results were not affected after the omission of subject with diabetes or with a history of CVD. Tables S4–S5 show baseline characteristics of study participants respectively according to LDL-C concentrations at baseline according to gender in KSHS cohort.

### 3.3. Association between Low-Density Lipoprotein Cholesterol and All-Cause, CVD, and Cancer Mortality in Korean Genome and Epidemiology Study

In the validation cohort (KoGES), 2338 deaths (1823 from cancer and 199 from CVD) occurred during follow-up of (mean ± SD) 8.57 ± 2.59 years. Table S6 represents baseline characteristics of KoGES participants according to baseline LDL-C concentrations. There were 2338 deaths (1.28%) during follow up in KoGES. Of these deaths, 199 deaths were due to CVD (121 in men and 78 in women) and 675 deaths were due to cancer (482 deaths in men and 193 deaths in women) in KoGES. The results of cox regression models showing risk of all-cause mortality, CVD mortality, and cancer mortality according to baseline LDL-C concentrations are shown in Table 5, Table 6 and Table 7, respectively. In the lowest LDL-C group (LDL < 70 mg/dL) comparing reference group (LDL 100–129 mg/dL), the adjusted HR (95% CIs) were 1.81 (1.44–2.28) for all-cause mortality, 1.93 (0.81–4.61) for CVD mortality, and 1.24 (0.95–1.63) for cancer mortality after adjustment for age, BMI, smoking status, alcohol intake, house income, marriage status, hypertension, diabetes and HDL-C concentrations. Similar to the results obtained within KHSH, the association between the lowest LDL-C and higher risk of mortality was more prominent in men than in women. Additionally, a U-shaped association between LDL-C concentrations and CVD mortality in men were observed with a nadir at 100–129 mg/dL in KoGES data.

## 4. Discussion

Our novel results show that low levels of LDL-C (<70 mg/dL) were associated with increased risk of CVD mortality, cancer mortality, and even all-cause mortality especially in men who were not treated with lipid lowering therapy. The finding of increased CVD mortality in men with low levels of LDL-C (<70 mg/dL) was observed in both different cohorts even though it showed a U shape. In this study, we were able to take account of multiple confounders and the young age of the cohort helped decrease the influence of potential reverse causality between clinically relevant outcomes and low levels of plasma LDL-C concentrations. Additionally, we excluded subjects who died within 3 years of follow up to avoid the possibility of reverse causality. Furthermore, to validate these associations, we then analyzed other dataset from a large population-based cohort study with government funding, named the Korean genome and epidemiology study (KoGES) which consists of community-dwellers aged ≥40 years at baseline.

We chose the third LDL-C group (i.e., LDL-C 100–129 mg/dL) as the reference group, because as indicated above this group contained the mean LDL-C concentration for the Korean population as measured in the Korean National Health and Nutrition Examination Survey during 1998 to 2010 [15]. As we expected, the highest category of LDL-C (≥160 mg/dL or ≥130 mg/dL in KoGES) was associated with increased risk of CVD mortality. However, the lowest LDL-C concentration category (LDL-C <70 mg/dL) also showed higher risk of CVD mortality compared to the reference group.

In line with our findings, another recent study also presented that whereas low LDL-C (<70 mg/dL) was not associated with protective effects on CVD outcome, low hs-CRP appeared to be associated with reduced risk of incident CVD and CVD mortality in high risk population [18]. These findings provide a paradoxical contradiction to the traditional LDL-C hypothesis; a lower CVD and all-cause mortality in lower LDL-C levels. It suggests the possibility that lower LDL-C concentration itself may not be a crucial factor for health outcome and other factor such as inflammatory process may have more important role in health outcome. However, considering the known strong association lowering LDL-C levels and better CV outcome, our finding indicates potential higher risk of poor health outcome in subjects who have too lower level of LDL-C although they do not take lipid lowering agents.

Associations between lower levels of LDL-C and poor health outcome have been reported in some, but not all, prior studies. Observational cohort studies have revealed that people with low total cholesterol levels (e.g., total cholesterol < 154.4 mg/dL) have increased risk of subsequent death in some cancers, respiratory diseases, and other non-medical causes than people with high baseline cholesterol levels [4]. A recent systematic review of 19 cohort studies including more than 68,000 elderly people showed that CVD mortality was highest in the lowest LDL-C quartile group [19]. However, these studies included participants who were taking lipid-lowering agents and who had other co-morbidities which may have influenced outcomes. Our study has excluded all subjects who were taking any lipid-lowering therapy at baseline in order to investigate the direct association between low levels of LDL-C and mortality outcomes. We demonstrated an increase in any cause of mortality outcomes in the lowest LDL-C concentration group especially in men. The finding of increased risk of mortality in men with low level of LDL-C was similar when we even excluded subjects who have history of diabetes and CVD at baseline. We additionally confirmed this phenomenon in another validation cohort. Our finding provide evidence supporting the ‘lipid paradox’, suggesting that too lower level of cholesterol concentrations do not always confer protective effects on mortality outcomes in the healthy population who does not take lipid-lowering agents.

While the exact mechanism remains to be elucidated, several possibilities could explain our findings. Firstly, a low LDL-C concentration increases susceptibility to fatal disease. Some experiments have shown that LDL-C binds to and inactivates a broad range of microorganisms and toxic products which might be a possible causal factor of CVD and cancer [20,21]. Furthermore, a common mechanism may operate that links low LDL-C concentration to different disease states. Links between low LDL-C and death from different diseases, only seems plausible if low LDL-C concentration is a marker for another phenomenon and to this effect although it is pure speculation, we and others have suggested that dysbiosis and altered bile acid metabolism [22,23,24,25] could provide that common link.

There are strengths and limitations of our study that should be considered in the interpretation of these controversial data. A number of 347,971 relatively young subjects (mean age 39.6 years) (57.4% men) were studied in a retrospective cohort study design over a median follow up of almost 6 years and data on cardiovascular mortality in men validated in another independent cohort. Additionally, we have excluded the data from individuals who were identified at baseline and who subsequently died during the first three years of follow up. These factors limit the possibility of reverse causality explaining our findings. However, since we excluded all subjects at baseline who were taking any lipid-lowering therapy, it is likely that subjects with extremely highest level of LDL-C have been excluded. Moreover, the weaknesses of our study design is that treatment with LDL-C lowering therapy during the period of follow up is not available, although given what is known about the benefit of statins, treatment with statins would decrease CVD and misclassification bias would operate to bias our results towards the null. Additionally, the fact that the numbers of deaths, especially cardiovascular mortality, are relatively low may attenuate the causal relationship between LDL cholesterol and mortality. We could not measure some specific lipoproteins such as small dense LDL and lipoprotein, which may explain this phenomenon. Another limitation of our study is that the sample was limited to Korean, relatively young-aged participants, and it is uncertain whether the findings are applicable to other ethnic groups.

## 5. Conclusions

We demonstrated that low levels of LDL-C concentration (<70 mg/dL) was not associated with protective effects on overall mortality in relatively healthy Korean adults who do not take lipid lowering agents. While men with the lowest levels of LDL-C concentration (<70 mg/dL) are at risk of increased all-cause, cancer, and even CVD mortality, and even though the association between LDL-C concentration and CVD mortality was U-shaped in men, the lowest levels of LDL-C concentration were significantly and independently associated with increased risk of CVD mortality. These findings suggest that more attention might be needed for subjects with no statin-induced decrease in LDL-C concentrations. Further large-scale, population-based research with long-term follow up is warranted in other ethnic groups to re-evaluate the relationship between low levels of LDL-C and mortality outcomes.

## Figures and Tables

**Table 1:** Baseline characteristics of participants according to LDL-C concentrations in Kangbuk Samsung Health Study.

Characteristics	Overall	LDL-C (mg/dL)					*p* for Trend
		<70	70–99	100–129	130–159	≥160	
Number	347,971	18,298	97,660	131,879	73,614	26,520	
Age (years)	39.5 (9.8)	37.2 (9.4)	37.6 (9.0)	39.6 (9.6)	41.4 (10.1)	42.7 (10.5)	<0.001
BMI (kg/m^2^)	23.4 (3.1)	21.8 (3.0)	23.3 (2.9)	23.5 (3.0)	24.5 (3.0)	25.1 (3.0)	<0.001
Systolic BP (mmHg)	113.8 (14.4)	110.2 (14.2)	110.9 (13.8)	114.0 (14.2)	116.6(14.3)	118.20 (14.7)	<0.001
Diastolic BP (mmHg)	73.3 (10.1)	70.6 (10.0)	71.3 (9.8)	73.5 (10.0)	75.4 (10.1)	76.4 (10.2)	<0.001
Laboratory							
Fasting glucose (mg/dL)	94.8 (16.5)	93.2 (16.5)	92.7 (13.9)	94.6 (15.6)	96.6 (18.0)	99.1 (22.4)	<0.001
Total cholesterol (mg/dL)	194.6 (34.9)	144.4 (26.8)	166.6 (18.7)	193.6 (17.9)	222.7 (18.0)	259.6 (25.1)	<0.001
HDL-C (mg/dL)	55.8 (13.0)	58.2 (15.8)	57.4 (14.0)	55.3 (12.8)	54.2 (11.6)	54.7 (11.2)	<0.001
Triglycerides (mg/dL)	101 (71–150)	73 (53–114)	80 (59–118)	102 (74–148)	123 (91–171)	139 (104–186)	<0.001
Smoking status (%)							
Never smoker	55.5	64.4	63.5	54.9	47.4	45.2	<0.001
Former smoker	16.8	12.6	13.4	17.2	20.2	20.6	<0.001
Current smoker	27.6	22.8	22.9	27.8	32.3	34.1	<0.001
Alcohol intake (%)							
0 g/day	33.6	37.7	36.8	33.1	30.3	30.7	<0.001
10 g/day	49.7	46.7	49.1	50.4	50.3	48.7	<0.001
20 g/day	16.6	15.4	13.9	16.4	19.3	20.5	<0.001
Regular exercise (%) ^1^	15.7	15.2	15.4	16.1	15.9	14.6	0.969
Hx of Hypertension (%)	7.6	6.3	5.6	7.3	9.8	11.6	<0.001
Hx of Diabetes mellitus (%)	2.3	3.1	2.0	2.1	2.4	2.7	<0.001
Hx of coronary artery disease (%)	3.6	4.6	3.6	3.4	3.6	4.0	0.159
Diabetes (%)	3.7	4.2	2.8	3.4	4.4	6.0	<0.001
Hypertension (%)	15.8	11.6	11.3	15.5	20.5	23.6	<0.001
Medication for diabetes (%)	1.6	2.5	1.4	1.5	1.6	1.6	0.029
Medication for hypertension (%)	5.3	5.2	4.1	5.2	6.5	7.2	<0.001
Higher education (%) ^2^	72.3	70.3	72.5	72.7	72.5	70.2	0.194

Data are mean (standard deviation), median (interquartile range), or percentage. BMI = body mass index; BP = blood pressure; HDL-C = high-density lipoprotein cholesterol; LDL-C = low-density lipoprotein cholesterol. ^1^ ≥3 time per week; ^2^ Above college graduate. SI unit Conversion (Multiply the conversion factor): Glucose, 0.0555 (mmol/L); total cholesterol, 0.0259 (mmol/L); HDL-C, 0.0259 (mmol/L); LDL-C, 0.0259 (mmol/L); triglyceride, 0.0113 (mmol/L).

**Table 2:** Risk of all-cause mortality according to baseline LDL-C levels excluding subjects who died within 3 years of follow up in Kangbuk Samsung Health Study.

LDL-C (mg/dL)	Person-Years	Number of Events	Mortality Rate (10,000 Person-Year)	Age-Adjusted HR (95% CI)	Multivariate HR (95% CI)		
					Model 1	Model 2	Model 3
Total (*n* = 347,322)							
LDL < 70	102,558.00	113	11	2.21	2.07	1.94	1.95
				(1.80–2.71)	(1.68–2.54)	(1.54–2.45)	(1.55–2.47)
LDL 70–99	548,590.40	323	5.8	1.16	1.16	1.14	1.15
				(1.01–1.34)	(1.01–1.34)	(0.97–1.34)	(0.98–1.35)
LDL 100–129	756,557.60	488	6.4	1.00 (reference)	1.00 (reference)	1.00 (reference)	1.00 (reference)
LDL 130–159	413,829.30	312	7.5	0.96	0.96	0.97	0.95
				(0.83–1.10)	(0.83–1.10)	(0.82–1.13)	(0.81–1.12)
LDL ≥ 160	139,579.80	143	10.2	1.16	1.19	1.18	1.15
				(0.96–1.40)	(0.99–1.44)	(0.96–1.46)	(0.93–1.42)
*p* for trend				0.001	0.001	0.003	0.001
Men (*n* = 199,195)							
LDL < 70	43,175.10	89	20.6	2.37	2.21	2.06	2.07
				(1.87–3.00)	(1.75–2.80)	(1.58–2.69)	(1.58–2.70)
LDL 70–99	261,503.70	221	8.5	1.17	1.15	1.11	1.12
				(0.99–1.38)	(0.97–1.36)	(0.92–1.34)	(0.92–1.34)
LDL 100–129	462,384.70	351	7.6	1.00 (reference)	1.00 (reference)	1.00 (reference)	1.00 (reference)
LDL 130–159	290,047.80	231	8	1	0.99	0.98	0.97
				(0.85–1.18)	(0.84–1.17)	(0.81–1.18)	(0.80–1.17)
LDL ≥ 160	99,505.80	99	9.9	1.27	1.24	1.29	1.25
				(1.01–1.58)	(0.99–1.55)	(1.01–1.65)	(0.98–1.61)
*p* for trend				0.001	0.001	0.032	0.016
Women (*n* = 148,127)							
LDL < 70	59,382.90	24	4	1.6	1.54	1.53	1.56
				(1.04–2.48)	(0.99–2.39)	(0.94–2.51)	(0.95–2.55)
LDL 70–99	287,086.70	102	3.5	1.2	1.15	1.2	1.21
				(0.91–1.52)	(0.89–1.50)	(0.90–1.59)	(0.91–1.62)
LDL 100–129	294,173.00	137	4.7	1.00 (reference)	1.00 (reference)	1.00 (reference)	1.00 (reference)
LDL 130–159	123,781.60	81	6.5	0.9	0.91	0.97	0.96
				(0.68–1.19)	(0.69–1.20)	(0.72–1.30)	(0.71–1.29)
LDL ≥ 160	40,074.00	44	11	1.16	1.15	1.01	0.98
				(0.82–1.63)	(0.82–1.63)	(0.68–1.50)	(0.66–1.47)
*p* for trend				0.12	0.172	0.103	0.07
*p* for interaction by gender				0.507	0.608	0.487	0.495

CI = confidence intervals; HDL-C = high-density lipoprotein cholesterol; HR = hazard ratio; LDL-C = low-density lipoprotein cholesterol. Model 1: Adjusted for age, body mass index, smoking status, alcohol intake, regular exercise. Model 2: Model 1 + education level, hypertension, diabetes, history of coronary artery disease. Model 3: Model 2 + HDL-C.

**Table 3:** Risk of cardiovascular disease mortality according to baseline LDL-C levels excluding subjects who died within 3 years of follow up in Kangbuk Samsung Health Study.

LDL-C (mg/dL)	Person-Years	Number of Events	Mortality Rate (10,000 Person-Year)	Age-Adjusted HR (95% CI)	Multivariate HR (95% CI)		
					Model 1	Model 2	Model 3
Total (*n* = 347,322)							
LDL < 70	102,558.00	14	1.3	1.99	1.83	2.03	2.02
				(1.12–3.55)	(1.02–3.27)	(1.12–3.67)	(1.11–3.64)
LDL 70–99	548,590.40	34	0.6	0.9	0.91	0.93	0.92
				(0.59–1.36)	(0.60–1.38)	(0.59–1.45)	(0.59–1.43)
LDL 100–129	756,557.60	67	0.8	1.00 (reference)	1.00 (reference)	1.00 (reference)	1.00 (reference)
LDL 130–159	413,829.30	53	1.2	1.17	1.15	1.17	1.19
				(0.81–1.68)	(0.80–1.66)	(0.79–1.73)	(0.80–1.75)
LDL ≥ 160	139,579.80	20	1.4	1.16	1.16	1.05	1.08
				(0.70–1.92)	(0.70–1.92)	(0.60–1.84)	(0.61–1.89)
*p* for trend				0.957	0.93	0.678	0.764
Men (*n* = 199,195)							
LDL < 70	43,175.10	10	2	1.91	1.77	2.01	1.99
				(0.97–3.77)	(0.89–3.52)	(0.99–4.04)	(0.99–4.02)
LDL 70–99	261,503.70	25	0.9	0.94	0.94	1.01	1
				(0.58–1.53)	(0.58–1.53)	(0.60–1.69)	(0.59–1.68)
LDL 100–129	462,384.70	49	1.1	1.00 (reference)	1.00 (reference)	1.00 (reference)	1.00 (reference)
LDL 130–159	290,047.80	35	1.2	1.09	1.05	1.07	1.08
				(0.71–1.68)	(0.68–1.62)	(0.66–1.72)	(0.67–1.75)
LDL ≥ 160	99,505.80	15	1.6	1.37	1.27	1.2	1.23
				(0.77–2.45)	(0.71–2.28)	(0.63–2.29)	(0.64–2.37)
*p* for trend				0.889	0.983	0.612	0.674
Women (*n* = 148,127)							
LDL < 70	290,047.80	4	0.6	2.47	2.35	2.44	2.41
				(0.83–7.34)	(0.79–7.02)	(0.81–7.35)	(0.80–7.29)
LDL 70–99	287,086.70	9	0.3	0.91	0.87	0.82	0.81
				(0.41–2.03)	(0.39–1.94)	(0.35–1.91)	(0.35–1.90)
LDL 100–129	294,173.00	18	0.6	1.00 (reference)	1.00 (reference)	1.00 (reference)	1.00 (reference)
LDL 130–159	123,781.60	18	1.5	1.34	1.37	1.35	1.36
				(0.70–2.59)	(0.71–2.65)	(0.68–2.66)	(0.69–2.68)
LDL ≥ 160	40,074.00	5	1.2	0.85	0.87	0.74	0.75
				(0.31–2.30)	(0.32–2.35)	(0.25–2.21)	(0.25–2.27)
*p* for trend				0.747	0.888	0.751	0.798
*p* for interaction by gender				0.808	0.798	0.76	0.759

CI = confidence intervals; HDL-C = high-density lipoprotein cholesterol; HR = hazard ratio; LDL-C = low-density lipoprotein cholesterol. Model 1: Adjusted for age, body mass index, smoking status, alcohol intake, regular exercise. Model 2: Model 1 + education level, hypertension, diabetes, history of coronary artery disease. Model 3: Model 2 + HDL-C.

**Table 4:** Risk of cancer mortality according to baseline LDL-C levels excluding subjects who died within 3 years of follow up in Kangbuk Samsung Health Study.

LDL-C (mg/dL)	Person-Years	Number of Events	Mortality Rate (10,000 Person-Year)	Age-Adjusted HR (95% CI)	Multivariate HR (95% CI)		
					Model 1	Model 2	Model 3
Total (*n* = 347,322)							
LDL < 70	102,558.00	50	4.8	2.01	1.95	2.05	2.06
				(1.48–2.73)	(1.43–2.65)	(1.45–2.88)	(1.46–2.90)
LDL 70–99	548,590.40	159	2.8	1.17	1.19	1.2	1.21
				(0.96–1.44)	(0.98–1.46)	(0.96–1.51)	(0.97–1.52)
LDL 100–129	756,557.60	238	3.1	1.00 (reference)	1.00 (reference)	1.00 (reference)	1.00 (reference)
LDL 130–159	413,829.30	150	3.6	0.94	0.93	0.97	0.96
				(0.77–1.16)	(0.76–1.14)	(0.77–1.22)	(0.76–1.20)
LDL ≥ 160	139,579.80	78	5.5	1.3	1.3	1.31	1.27
				(1.00–1.68)	(1.01–1.69)	(0.98–1.74)	(0.95–1.70)
*p* for trend				0.033	0.03	0.058	0.033
Men (n = 199,195)							
LDL < 70	43,175.10	43	9.9	2.41	2.33	2.42	2.42
				(1.72–3.38)	(1.66–3.26)	(1.66–3.53)	(1.66–3.53)
LDL 70–99	261,503.70	111	4.2	1.24	1.24	1.19	1.19
				(0.98–1.58)	(0.97–1.57)	(0.91–1.56)	(0.91–1.57)
LDL 100–129	462,384.70	166	3.6	1.00 (reference)	1.00 (reference)	1.00 (reference)	1.00 (reference)
LDL 130–159	290,047.80	109	3.8	1	0.98	1	0.99
				(0.78–1.27)	(0.77–1.25)	(0.76–1.31)	(0.76–1.30)
LDL ≥ 160	99,505.80	53	5.3	1.43	1.38	1.37	1.36
				(1.05–1.95)	(1.01–1.88)	(0.97–1.95)	(0.96–1.93)
*p* for trend				0.025	0.02	0.06	0.047
Women (*n* = 148,127)							
LDL < 70	290,047.80	7	1.1	0.83	0.86	0.97	0.99
				(0.38–1.81)	(0.39–1.87)	(0.42–2.27)	(0.43–2.32)
LDL 70–99	287,086.70	48	1.6	1	1.03	1.17	1.19
				(0.69–1.44)	(0.71–1.49)	(0.78–1.75)	(0.79–1.79)
LDL 100–129	294,173.00	72	2.4	1.00 (reference)	1.00 (reference)	1.00 (reference)	1.00 (reference)
LDL 130–159	123,781.60	41	3.3	0.91	0.89	0.98	0.97
				(0.62–1.34)	(0.61–1.31)	(0.64–1.49)	(0.63–1.48)
LDL ≥ 160	40,074.00	25	6.2	1.34	1.28	1.27	1.22
				(0.85–2.12)	(0.81–2.04)	(0.75–2.13)	(0.73–2.06)
*p* for trend				0.418	0.624	0.94	0.91
*p* for interaction by gender				0.271	0.305	0.448	0.46

CI = confidence intervals; HDL-C = high-density lipoprotein cholesterol; HR = hazard ratio; LDL-C = low-density lipoprotein cholesterol. Model 1: Adjusted for age, body mass index, smoking status, alcohol intake, regular exercise. Model 2: Model 1 + education level, hypertension, diabetes, history of coronary artery disease. Model 3: Model 2 + HDL-C.

**Table 5:** Risk of all-cause mortality according to baseline LDL-C levels excluding subjects who died within 3 years of follow up in KoGES data.

LDL-C (mg/dL)	Person-Years	Number of Events	Mortality Rate (10,000 Person-Year)	Age-Adjusted HR (95% CI)	Multivariate HR (95% CI)		
					Model 1	Model 2	Model 3
Total (*n* = 182,943)							
LDL < 70	68,149	207	30.4	2.16	2.15	1.82	1.81
				(1.85–2.51)	(1.71–2.70)	(1.45–2.29)	(1.44–2.28)
LDL 70–99	357,358	565	15.8	1.28	1.45	1.35	1.35
				(1.15–1.43)	(1.23–1.70)	(1.15–1.59)	(1.15–1.59)
LDL 100–129	597,261	800	13.4	1.00 (reference)	1.00 (reference)	1.00 (reference)	1.00 (reference)
LDL 130–159	388,906	520	13.4	0.88	1.03	1.06	1.06
				(0.79–0.98)	(0.87–1.22)	(0.89–1.25)	(0.90–1.25)
LDL ≥ 160	157,296	246	15.6	0.94	1.05	1.08	1.08
				(0.81–1.08)	(0.84–1.31)	(0.86–1.35)	(0.87–1.36)
*p* for trend				<0.0001	<0.0001	<0.0001	0.0001
Men (*n* = 63,318)							
LDL < 70	33,865	174	51.4	2.06	2.17	1.83	1.83
				(1.73–2.45)	(1.69–2.79)	(1.42–2.36)	(1.42–2.36)
LDL 70–99	133,332	405	30.4	1.31	1.46	1.37	1.37
				(1.14–1.49)	(1.21–1.76)	(1.13–1.65)	(1.13–1.65)
LDL 100–129	210,838	487	23.1	1.00 (reference)	1.00 (reference)	1.00 (reference)	1.00 (reference)
LDL 130–159	124,980	258	20.6	0.9	0.97	0.98	0.98
				(0.78–1.05)	(0.78–1.21)	(0.79–1.22)	(0.79–1.22)
LDL ≥ 160	41,542	114	27.4	1.2	1.09	1.11	1.11
				(0.98–1.47)	(0.80–1.49)	(0.81–1.52)	(0.81–1.52)
*p* for trend				<0.0001	<0.0001	<0.0001	<0.0001
Women (*n* = 119,625)							
LDL < 70	34,284	33	9.6	1.29	1.48	1.3	1.3
				(0.90–1.85)	(0.83–2.64)	(0.73–2.33)	(0.73–2.32)
LDL 70–99	224,026	160	7.1	1.06	1.31	1.23	1.23
				(0.87–1.28)	(0.96–1.78)	(0.91–1.68)	(0.90–1.68)
LDL 100–129	386,423	313	8.1	1.00 (reference)	1.00 (reference)	1.00 (reference)	1.00 (reference)
LDL 130–159	263,926	262	9.9	0.96	1.24	1.28	1.28
				(0.81–1.13)	(0.95–1.63)	(0.98–1.68)	(0.98–1.68)
LDL ≥ 160	115,755	132	11.4	0.97	1.21	1.25	1.26
				(0.79–1.19)	(0.86–1.69)	(0.89–1.75)	(0.90–1.76)
*p* for trend				0.1964	0.8682	0.6045	0.5733

CI = confidence intervals; HDL-C = high-density lipoprotein cholesterol; HR = hazard ratio; LDL-C = low-density lipoprotein cholesterol. Model 1: Adjusted for age, body mass index, smoking status, alcohol intake, regular exercise. Model 2: Model 1 + education level, hypertension, diabetes, history of coronary artery disease. Model 3: Model 2 + HDL-C.

**Table 6:** Risk of cardiovascular disease mortality according to baseline LDL-C levels excluding subjects who died within 3 years of follow up in KOGES data.

LDL-C (mg/dL)	Person-Years	Number of Events	Mortality Rate (10,000 Person-Year)	Age-Adjusted HR (95% CI)	Multivariate HR (95% CI)		
					Model 1	Model 2	Model 3
Total (*n* = 180,804)							
LDL < 70	66,931	14	2.1	2	2.53	2	1.93
				(1.12–3.58)	(1.07–6.01)	(0.84–4.78)	(0.81–4.61)
LDL 70–99	353,873	39	1.1	1.21	2.07	1.88	1.86
				(0.81–1.81)	(1.14–3.74)	(1.04–3.42)	(1.03–3.37)
LDL 100–129	592,339	59	1	1.00 (reference)	1.00 (reference)	1.00 (reference)	1.00 (reference)
LDL 130–159	385,834	53	1.4	1.21	1.7	1.77	1.82
				(0.83–1.75)	(0.94–3.07)	(0.98–3.20)	(1.01–3.31)
LDL ≥ 160	155,906	34	2.2	1.74	2.65	2.83	3
				(1.14–2.65)	(1.37–5.14)	(1.46–5.49)	(1.54–5.81)
*p* for trend				0.2492	0.7319	0.3009	0.2016
Men (*n* = 62,001)							
LDL < 70	32,840	11	3.3	2.25	3.91	3.25	3.15
				(1.12–4.50)	(1.51–10.01)	(1.24–8.47)	(1.21–8.21)
LDL 70–99	130,856	30	2.3	1.64	2.78	2.6	2.55
				(0.99–2.74)	(1.32–5.84)	(1.23–5.47)	(1.21–5.38)
LDL 100–129	207,799	29	1.4	1.00 (reference)	1.00 (reference)	1.00 (reference)	1.00 (reference)
LDL 130–159	123,520	31	2.5	1.82	2.25	2.35	2.4
				(2.00–3.02)	(1.04–4.91)	(1.08–5.13)	(1.10–5.22)
LDL ≥ 160	40,912	20	4.9	3.57	3.46	3.7	3.85
				(2.02–6.31)	(1.39–8.63)	(1.49–9.24)	(1.54–9.60)
*p* for trend				0.0164	0.9761	0.6235	0.5223
Women (*n* = 118,803)							
LDL < 70	34,091	3	0.9	1.21	NA *	NA *	NA *
				(0.37–3.96)			
LDL 70–99	223,017	9	0.4	0.63	1.11	0.97	0.97
				(0.30–1.32)	(0.37–3.31)	(0.33–2.92)	(0.32–2.90)
LDL 100–129	384,541	30	0.8	1.00 (reference)	1.00 (reference)	1.00 (reference)	1.00 (reference)
LDL 130–159	262,314	22	0.8	0.83	1.12	1.21	1.24
				(0.48–1.43)	(0.45–2.83)	(0.48–3.05)	(0.49–3.13)
LDL ≥ 160	114,994	14	1.2	1.04	2	2.18	2.27
				(0.55–1.95)	(0.77–5.20)	(0.84–5.67)	(0.87–5.93)
*p* for trend				0.53	0.3287	0.1649	0.1363

CI = confidence intervals; HDL-C = high-density lipoprotein cholesterol; HR = hazard ratio; LDL-C = low-density lipoprotein cholesterol. Model 1: Adjusted for age, body mass index, smoking status, alcohol intake, regular exercise. Model 2: Model 1 + education level, hypertension, diabetes, history of coronary artery disease. Model 3: Model 2 + HDL-C. * can not calculate due to low number of event.

**Table 7:** Risk of cancer mortality according to baseline LDL-C levels excluding subjects who died within 3 years of follow up in KoGES data.

LDL-C (mg/dL)	Person-Years	Number of Events	Mortality Rate (10,000 Person-Year)	Age-Adjusted HR (95% CI)	Multivariate HR (95% CI)		
					Model 1	Model 2	Model 3
Total (*n* = 182,428)							
LDL < 70	67,784	145	21.4	1.92	1.31	1.24	1.24
				(1.60–2.29)	(1.01–1.72)	(0.95–1.62)	(0.95–1.63)
LDL 70–99	356,508	449	12.6	1.26	1.07	1.04	1.04
				(1.12–1.43)	(0.90–1.26)	(0.88–1.23)	(0.88–1.23)
LDL 100–129	596,054	640	10.7	1.00 (reference)	1.00 (reference)	1.00 (reference)	1.00 (reference)
LDL 130–159	388,202	421	10.8	0.9	0.97	0.98	0.97
				(0.80–1.02)	(0.82–1.14)	(0.83–1.15)	(0.83–1.15)
LDL ≥ 160	156,791	168	10.7	0.82	0.89	0.9	0.9
				(0.69–0.97)	(0.71–1.13)	(0.71–1.14)	(0.71–1.13)
*p* for trend				<0.0001	<0.0001	0.0022	0.0021
Men (*n* = 62,993)							
LDL < 70	33,550	119	35.5	1.82	1.41	1.34	1.34
				(1.48–2.24)	(1.05–1.91)	(0.99–1.82)	(0.99–1.82)
LDL 70–99	132,685	310	23.4	1.29	1.09	1.06	1.06
				(1.11–1.50)	(0.88–1.34)	(0.86–1.31)	(0.86–1.31)
LDL 100–129	210,008	379	18	1.00 (reference)	1.00 (reference)	1.00 (reference)	1.00 (reference)
LDL 130–159	124,852	239	19.1	1.07	1.14	1.14	1.14
				(0.91–1.26)	(0.92–1.40)	(0.93–1.41)	(0.92–1.41)
LDL ≥ 160	41,233	66	16	0.9	0.87	0.88	0.87
				(0.70–1.17)	(0.62–1.23)	(0.62–1.24)	(0.62–1.24)
*p* for trend				<0.0001	0.0949	0.1915	0.1837
Women (*n* = 119,435)							
LDL < 70	34,234	26	7.6	1.22	0.96	0.9	0.9
				(0.82–1.82)	(0.52–1.77)	(0.48–1.66)	(0.49–1.67)
LDL 70–99	223,823	139	6.2	1.04	1	0.98	0.98
				(0.85–1.28)	(0.75–1.33)	(0.73–1.30)	(0.74–1.30)
LDL 100–129	386,046	261	6.8	1.00 (reference)	1.00 (reference)	1.00 (reference)	1.00 (reference)
LDL 130–159	263,350	182	6.9	0.86	0.82	0.83	0.833
				(0.71–1.04)	(0.63–1.07)	(0.64–1.09)	(0.64–1.09)
LDL ≥ 160	115,558	102	8.8	0.99	0.98	1	0.99
				(0.79–1.25)	(0.71–1.35)	(0.72–1.37)	(0.72–1.37)
*p* for trend				0.0994	0.4654	0.6899	0.6713

CI = confidence intervals; HDL-C = high-density lipoprotein cholesterol; HR = hazard ratio; LDL-C = low-density lipoprotein cholesterol. Model 1: Adjusted for age, body mass index, smoking status, alcohol intake, regular exercise. Model 2: Model 1 + education level, hypertension, diabetes, history of coronary artery disease. Model 3: Model 2 + HDL-C.

## Supplementary Materials

The following are available online at https://www.mdpi.com/2077-0383/8/10/1571/s1, Table S1: Risk of death from all causes according to baseline LDL excluding subjects who died within 3 years of follow up and excluding subjects with existing diabetes or cardiovascular disease at baseline, Table S2: Risk of death from cardiovascular disease according to baseline LDL excluding subjects who died within 3 years of follow up and excluding subjects with existing diabetes or cardiovascular disease at baseline, Table S3: Risk of death from cancer according to baseline LDL excluding subjects who died within 3 years of follow up and excluding subjects with existing diabetes or cardiovascular disease at baseline; Table S4. Baseline characteristics of men according to LDL-C concentrations in KSHS (Men), Table S5. Baseline characteristics of men according to LDL-C concentrations in KSHS (Women), Table S6. Baseline characteristics of participants according to LDL-C concentrations in KoGES.
