# Supplementary material for: Low Levels of Low-Density Lipoprotein Cholesterol and Mortality Outcomes in Non-Statin Users
Source: J Clin Med. 2019 Oct 1;8(10):1571. doi: 10.3390/jcm8101571 (PMC6832139; doi:10.3390/jcm8101571)
Supplement: Supplementary file 1 [file jcm-08-01571-s001.pdf]

Table S1. Risk of death from all causes according to baseline LDL excluding subjects who died within 3 years of follow up and excluding subjects with existing diabetes or cardiovascular disease at baseline in KSHS.

LDL-C (mg/dL)	Person-years	Number of events	Mortality rate (10,000 person-year)	Age-adjusted HR (95% CI)	Multivariate HR (95% CI)		
					Model 1	Model 2	Model 3
Men (n=184,055)							
LDL <70	38,515.5	63	16.4	2.44 (1.86-3.21)	2.24 (1.70-2.95)	2.31 (1.71-3.12)	2.31 (1.71-3.12)
LDL 70-99	242,959.5	175	7.2	1.20 (0.99-1.45)	1.17 (0.97-1.42)	1.17 (0.95-1.44)	1.17 (0.95-1.45)
LDL 100-129	431,872.4	279	6.5	1.00 (reference)	1.00 (reference)	1.00 (reference)	1.00 (reference)
LDL 130-159	269,988.8	183	6.8	0.99 (0.82-1.19)	0.97 (0.81-1.17)	0.98 (0.80-1.20)	0.97 (0.79-1.19)
LDL ≥160	91,228.0	79	8.7	1.25 (0.97-1.60)	1.21 (0.94-1.56)	1.24 (0.94-1.63)	1.20 (0.91-1.58)
p for trend				0.001	0.003	0.008	0.004
Women (n=138,772)							
LDL <70	56,304.1	21	3.7	1.67 (1.04-2.67)	1.57 (0.98-2.52)	1.50 (0.89-2.53)	1.53 (0.91-2.57)
LDL 70-99	273,915.1	90	3.3	1.20 (0.91-1.58)	1.18 (0.89-1.56)	1.15 (0.85-1.56)	1.17 (0.87-1.58)
LDL 100-129	277,097.2	117	4.2	1.00 (reference)	1.00 (reference)	1.00 (reference)	1.00 (reference)
LDL 130-159	113,227.7	64	5.7	0.86 (0.63-1.17)	0.86 (0.63-1.17)	0.90 (0.65-1.24)	0.89 (0.65-1.23)
LDL ≥160	35,096.9	34	9.7	1.12 (0.76-1.65)	1.12 (0.76-1.64)	0.88 (0.56-1.37)	0.85 (0.54-1.34)
p for trend				0.058	0.091	0.048	0.033

CI = confidence intervals; HR = hazard ratio; LDL-C = low-density lipoprotein cholesterol. Cox proportional hazard models were used to estimate ORs and 95 percent confidence intervals (95% CIs). Model 1: Adjusted for age, body mass index, smoking status, alcohol intake, regular exercise. Model 2: Model 1 + education level, hypertension Model 3: Model 2 + HDL-C.

Table S2. Risk of death from cardiovascular disease according to baseline LDL excluding subjects who died within 3 years of follow up and excluding subjects with existing diabetes or cardiovascular disease at baseline in KSHS.

LDL-C (mg/dL)	Person-years	Number of events	Mortality rate (10,000 person-year)	Age-adjusted HR (95% CI)	Multivariate HR (95% CI)		
					Model 1	Model 2	Model 3
Men (n=184,055)							
LDL <70	38,515.5	8	2.1	2.40 (1.12-5.17)	2.25 (1.04-4.87)	2.78 (1.27-6.13)	2.77 (1.26-6.10)
LDL 70-99	242,959.5	16	0.7	0.85 (0.47-1.53)	0.86 (0.47-1.54)	1.04 (0.56-1.91)	1.03 (0.56-1.89)
LDL 100-129	431,872.4	36	0.8	1.00 (reference)	1.00 (reference)	1.00 (reference)	1.00 (reference)
LDL 130-159	269,988.8	24	0.9	1.00 (0.60-1.68)	0.95 (0.57-1.59)	0.99 (0.57-1.73)	1.01 (0.57-1.77)
LDL ≥160	91,228.0	14	1.5	1.71 (0.92-3.17)	1.53 (0.82-2.86)	1.41 (0.70-2.82)	1.46 (0.72-2.96)
p for trend				0.690	0.892	0.500	0.6562
Women (n=138,772)							
LDL <70	56,304.1	4	0.7	4.06 (1.30-12.72)	3.56 (1.13-11.25)	3.62 (1.14-11.49)	3.57 (1.12-11.42)
LDL 70-99	273,915.1	8	0.3	1.24 (0.50-3.05)	1.14 (0.46-2.81)	1.02 (0.40-2.60)	1.01 (0.39-2.59)
LDL 100-129	277,097.2	12	0.4	1.00 (reference)	1.00 (reference)	1.00 (reference)	1.00 (reference)
LDL 130-159	113,227.7	15	1.3	1.68 (0.79-3.61)	1.74 (0.81-3.73)	1.52 (0.70-3.32)	1.53 (0.70-3.33)
LDL ≥160	35,096.9	3	0.9	0.79 (0.22-2.82)	0.84 (0.24-2.98)	0.53 (0.12-2.40)	0.54 (0.12-2.44)
p for trend				0.403	0.604	0.349	0.377

CI = confidence intervals; HR = hazard ratio; LDL-C = low-density lipoprotein cholesterol. Cox proportional hazard models were used to estimate ORs and 95 percent confidence intervals (95% CIs). Model 1: Adjusted for age, body mass index, smoking status, alcohol intake, regular exercise. Model 2: Model 1 + education level, hypertension. Model 3: Model 2 + HDL-C.

Table S3. Risk of death from cancer according to baseline LDL excluding subjects who died within 3 years of follow up and excluding subjects with existing diabetes or cardiovascular disease at baseline in KSHS.

LDL-C (mg/dL)	Person-years	Number of events	Mortality rate (10,000 person-year)	Age-adjusted HR (95% CI)	Multivariate HR (95% CI)		
					Model 1	Model 2	Model 3
Men (n=184,055)							
LDL <70	38,515.5	33	8.6	2.64 (1.80-3.86)	2.51 (1.71-3.68)	2.80 (1.86-4.22)	2.81 (1.86-4.22)
LDL 70-99	242,959.5	92	3.8	1.31 (1.01-1.71)	1.30 (1.00-1.70)	1.30 (0.97-1.75)	1.30 (0.97-1.75)
LDL 100-129	431,872.4	134	3.1	1.00 (reference)	1.00 (reference)	1.00 (reference)	1.00 (reference)
LDL 130-159	269,988.8	93	3.4	1.04 (0.80-1.36)	1.01 (0.78-1.32)	1.04 (0.77-1.39)	1.02 (0.76-1.37)
LDL ≥160	91,228.0	43	4.7	1.41 (1.00-1.99)	1.34 (0.95-1.90)	1.32 (0.90-1.94)	1.30 (0.88-1.91)
p for trend				0.024	0.020	0.016	0.011
Women (n=138,772)							
LDL <70	56,304.1	5	0.9	0.66 (0.26-1.65)	0.69 (0.27-1.71)	0.65 (0.23-1.81)	0.66 (0.24-1.84)
LDL 70-99	273,915.1	43	1.6	0.97 (0.66-1.44)	1.01 (0.68-1.50)	1.05 (0.69-1.60)	1.07 (0.70-1.63)
LDL 100-129	277,097.2	65	2.3	1.00 (reference)	1.00 (reference)	1.00 (reference)	1.00 (reference)
LDL 130-159	113,227.7	35	3.1	0.89 (0.59-1.34)	0.86 (0.57-1.30)	0.94 (0.61-1.45)	0.93 (0.60-1.44)
LDL ≥160	35,096.9	20	5.7	1.27 (0.77-2.12)	1.20 (0.72-2.00)	1.17 (0.67-2.03)	1.13 (0.65-1.97)
p for trend				0.430	0.679	0.681	0.809

CI = confidence intervals; HR = hazard ratio; LDL-C = low-density lipoprotein cholesterol. Cox proportional hazard models were used to estimate ORs and 95 percent confidence intervals (95% CIs). Model 1: Adjusted for age, body mass index, smoking status, alcohol intake, regular exercise. Model 2: Model 1 + education level, hypertension. Model 3: Model 2 + HDL-C.

Table S4. Baseline characteristics of men according to LDL-C concentrations in KSHS (Men).

Characteristics	Overall	LDL-C (mg/dL)					*P* for trend
		~70	70-100	100-130	130-160	160~	
N	199,671	7,255	44,088	78,390	51,067	18,871	
Age (years)	39.5(9.5)	39.5(10.7)	38.6(9.7)	39.3(9.4)	40.1(9.3)	40.5(9.1)	<0.001
BMI (kg/m^2)	24.4(2.9)	23.3(3.1)	23.6(2.9)	24.3(2.8)	25.0(2.8)	25.5(2.8)	<0.001
Systolic BP (mmHg)	117.5(13.1)	117.3(13.6)	116.5(12.9)	117.3(13.0)	118.3(13.3)	118.9(13.4)	<0.001
Diastolic BP (mmHg)	76.3(9.5)	75.8(9.6)	75.5(9.3)	76.2(9.5)	76.9(9.6)	77.5(9.8)	<0.001
Laboratory							
Fasting glucose(mg/dl)	96.7(17.8)	98.0(21.3)	95.7(16.4)	96.3(16.9)	97.3(18.3)	99.1(21.5)	<0.001
Total cholesterol (mg/dl)	199.0(34.6)	147.9(34.7)	166.7(20.1)	192.9(18.2)	221.8(18.0)	257.9(24.1)	<0.001
HDL-C (mg/dl)	52.1(11.4)	53.0(15.6)	52.6(12.8)	51.8(11.1)	51.8(10.2)	52.7(10.0)	<0.001
Triglycerides (mg/dl)	123(87-177)	102(67-210)	102(73-160)	119(86-171)	135(100-184)	148(112-195)	<0.001
Smoking status (%)							
Never smoker	28.5	26.6	29.9	29.3	27.3	25.9	<0.001
Former smoker	26.7	25.0	25.1	26.7	27.7	27.8	<0.001
Current smoker	44.8	48.4	45.0	44.0	44.9	46.3	0.959
Alcohol intake (%)							
0g/day	15.3	14.4	15.1	15.3	15.6	16.1	<0.001
10g/day	57.9	53.4	57.9	59.0	57.7	56.1	0.521
20g/day	26.8	32.3	27.1	25.8	26.8	27.9	0.009
Regular exercise (%)[1]	16.3	17.6	17.5	16.7	15.6	14.1	<0.001
Hx of Hypertension (%)	8.99	11.0	8.29	8.43	9.67	10.4	<0.001
Hx of Diabetes mellitus (%)	2.76	5.64	3.12	2.57	2.45	2.45	<0.001
Hx of coronary artery disease (%)	3.34	5.38	3.71	3.18	3.01	3.25	<0.001
Diabetes(%)	4.61	7.91	4.47	4.16	4.56	5.70	0.729
Hypertension(%)	19.9	21.0	17.8	19.1	21.7	23.1	<0.001
Diet cholesterol							
Higher education (%)	79.8	73.5	78.1	80.3	81.1	80.7	<0.001

BMI = body mass index; BP = blood pressure; HDL-C = high-density lipoprotein-cholesterol; LDL-C = low-density lipoprotein cholesterol. SI unit Conversion (Multiply the conversion factor): glucose, 0.0555 (mmol/L); total cholesterol, 0.0259 (mmol/L); HDL-C, 0.0259 (mmol/L); LDL-C, 0.0259 (mmol/L); triglyceride, 0.0113 (mmol/L). [1]≥ 3 time per week.

Table S5. Baseline characteristics of men according to LDL-C concentrations in KSHS (Women).

Characteristics	Overall	LDL-C (mg/dL)					*P* for trend
		~70	70-100	100-130	130-160	160~	
N	148,300	11,043	53,572	53,489	22,547	7,649	
Age (years)	39.7(10.2)	35.6(8.0)	36.9(8.4)	40.1(10.0)	44.5(11.2)	48.3(11.6)	<0.001
BMI (kg/m2)	22.1(3.1)	20.9(2.5)	21.3(2.7)	22.4(3.0)	23.5(3.3)	24.4(3.3)	<0.001
Systolic BP (mmHg)	108.9(14.5)	105.6(12.7)	106.4(12.9)	109.2(14.5)	112.9(16.1)	116.5(17.6)	<0.001
Diastolic BP (mmHg)	69.4(9.7)	67.3(8.9)	67.9(8.9)	69.7(9.6)	71.9(10.4)	73.8(10.8)	<0.001
Laboratory							
Fasting glucose(mg/dl)	92.2(14.1)	90.2(11.3)	90.4(10.9)	92.2(13.1)	95.2(17.6)	99.3(24.8)	<0.001
Total cholesterol (mg/dl)	188.8(34.4)	142.1(19.9)	166.6(17.6)	194.6(17.6)	225.0(18.0)	264.0(27.3)	<0.001
HDL-C (mg/dl)	60.9(13.5)	61.6(15.0)	61.5(13.8)	60.7(13.4)	59.9(12.8)	59.8(12.4)	<0.001
Triglycerides (mg/dl)	79(59-110)	63(49-86)	68(53-92)	82(63-112)	100(75-137)	118(88-158)	<0.001
Smoking status (%)							
Never smoker	91.9	89.3	91.3	92.4	93.1	92.9	<<0.001
Former smoker	3.67	4.55	3.90	3.52	3.26	3.14	<0.001
Current smoker	4.42	6.11	4.82	4.04	3.69	3.97	<0.001
Alcohol intake (%)							
0g/day	58.3	53.1	54.8	59.4	63.7	67.0	<0.001
10g/day	38.8	42.4	42.0	37.9	33.9	30.7	<0.001
20g/day	2.93	4.48	3.13	2.69	2.45	2.93	<0.001
Regular exercise (%)[1]	14.9	13.7	13.7	15.3	16.7	16.0	<0.001
Hx of Hypertension (%)	5.91	3.40	3.40	5.81	10.4	14.7	<0.001
Hx of Diabetes mellitus (%)	1.68	1.49	1.14	1.62	2.59	3.58	<0.001
Hx of coronary	4.07	4.11	3.57	3.92	4.92	6.07	<0.001
artery disease (%)	2.52	1.84	1.46	2.35	4.25	7.01	<0.001
Diabetes(%)	10.4	5.57	6.02	10.4	17.9	24.9	<0.001
Hypertension(%)	61.7	68.1	67.7	61.3	52.3	44.5	<0.001

BMI = body mass index; BP = blood pressure; HDL-C = high-density lipoprotein-cholesterol; LDL-C = low-density lipoprotein cholesterol. SI unit Conversion (Multiply the conversion factor): glucose, 0.0555 (mmol/L); total cholesterol, 0.0259 (mmol/L); HDL-C, 0.0259 (mmol/L); LDL-C, 0.0259 (mmol/L); triglyceride, 0.0113 (mmol/L). [1]≥ 3 time per week.

Table S6. Baseline characteristics of participants according to LDL-C concentrations in KoGES.

Characteristics	LDL (mg/dL)					p for trend
	<70	70-99	100-129	130-159	>160	
Number (%)	7,790 (4.37)	41,720 (22.80)	69,663 (38.08)	45,339 (24.78)	18,231 (9.97)	
Gender, men	3,981 (49.82)	15,524 (37.21)	24,528 (35.21)	14,533 (32.05)	4,752 (26.07)	<0.0001
Age (years)	53.08±9.39	51.99±8.94	52.93±8.69	54.36±8.44	55.56±8.00	<0.0001
BMI (kg/m2)	23.65±3.10	23.55±2.96	23.87±2.93	24.23±2.87	24.60±2.90	<0.0001
Systolic BP (mmHg)	123.56±16.34	121.33±15.93	122.22±15.86	123.65±15.87	125.20±16.22	<0.0001
Diastolic BP (mmHg)	76.86±10.66	75.75±10.43	76.42±10.32	77.35±10.21	78.24±10.30	<0.0001
Laboratory						
Glucose (mg/dl)	98.02±24.90	93.97±20.83	94.06±20.09	95.20±20.41	97.26±24.05	<0.0001
Total cholesterol (mg/dl)	139.72±19.29	164.53±16.98	191.33±16.22	220.20±15.84	257.40±23.00	<0.0001
HDL-C (mg/dl)	51.14±14.97	52.86±13.72	53.09±13.03	52.99±12.23	53.06±11.41	<0.0001
LDL-C (mg/dl)	58.82±10.10	87.78±8.16	114.86±8.50	142.83±8.41	178.56±17.86	<0.0001
Triglycerides (mg/dl)	148.76±95.28	119.49±74.62	116.92±63.86	121.91±58.97	128.89±57.32	<0.0001
Smoking status						<0.0001
Never smoker	4,686/7,910 (59.24)	29,329/41,338 (70.95)	50,228/69,010 (72.78)	33,480/44,949 (74.48)	14,024/18,073 (77.60)	
Former smoker	1469/7910 (18.57)	6,133/41,338 (14.84)	9,902/69,010 (14.35)	6,111/44,949 (13.60)	2,060/18,073 (11.40)	
Current smoker	1,755/7,910 (22.19)	5,876/41,338 (14.21)	8,880/69,010 (12.87)	5,358/44,949 (11.92)	1,989/18,073 (11.01)	
Alcohol intake						<0.0001
Never drinker	3,039/7,926 (38.34)	19,381/41,386 (46.83)	34,458/69,073 (49.89)	24,168/44,990 (53.72)	10,629/18,078 (58.80)	
Former drinker	426/7,926 (5.37)	1,824/41,386 (4.41)	2,813/69,073 (4.07%)	1,884/44,990 (4.19%)	744/18,078 (4.12%)	
Current drinker	4,461/7,926 (56.28)	20,181/41,386 (48.76)	31,802/69,073 (46.04)	18,938/44,990 (42.09)	6,705/18,078 (37.09)	
Regular exercise[1], No	3,898/7,447 (52.34)	19,879/39,408 (50.44)	33,304/65,843 (50.58)	21,834/42,730 (51.10)	9198/17,149 (53.64)	<0.0001
Hypertension	1,902/7,951 (23.92)	7,364/41,556 (17.72)	11,840/69,417 (17.06)	8,001/45,183 (17.71)	3,311/18,177 (18.22)	<0.0001
Diabetes mellitus	1131/7950 (14.23)	3,070/41,552 (7.39)	3,652/69,404 (5.26)	2,116/45,169 (4.68)	862/18,169 (4.74)	<0.0001
House income (monthly)						<0.0001
<1,500,000 won	1,817/6,441 (28.21)	8,293/33,724 (24.59)	14,191/56,055 (25.32)	10,336/36,297 (28.48)	4,521/14,355 (31.49)	
1,500,000 -3,000,000 won	2,161/6,441 (33.55)	11,057/33,724 (32.79)	18,332/56,055 (32.70)	11,657/36,297 (32.12)	4707/14,355 (32.79)	
3,000,000-6,000,000 won	2,052/6,441 (31.86)	11,815/33,724 (35.03)	19,356/56,055 (34.53)	11,622/36,297 (32.02)	4226/14,355 (29.44)	
>6,000,000 won	411/6,441 (6.38)	2,559/33,724 (7.59)	4,176/56,055 (7.45)	2,682/36,297 (7.39)	901/14,355 (6.28)	

BMI = body mass index; BP = blood pressure; HDL-C = high-density lipoprotein-cholesterol; LDL-C = low-density lipoprotein cholesterol. SI unit Conversion (Multiply the conversion factor): glucose, 0.0555 (mmol/L); total cholesterol, 0.0259 (mmol/L); HDL-C, 0.0259 (mmol/L); LDL-C, 0.0259 (mmol/L); triglyceride, 0.0113 (mmol/L). [1]≥ 1 time per week.
